# Young people’s preferences for HIV self-testing services in Nigeria: a qualitative analysis

**DOI:** 10.1186/s12889-020-10072-1

**Published:** 2021-01-07

**Authors:** Chisom Obiezu-Umeh, Titilola Gbajabiamila, Oliver Ezechi, Ucheoma Nwaozuru, Jason J. Ong, Ifeoma Idigbe, David Oladele, Adesola Z. Musa, Florida Uzoaru, Collins Airhihenbuwa, Joseph D. Tucker, Juliet Iwelunmor

**Affiliations:** 1 College for public Health and Social Justice, Saint Louis University, Saint Louis, MO USA; 2 Department of Clinical Sciences National Institute of Medical Research, Yaba, Lagos, Nigeria; 3 Department of Clinical Research, London School of Hygiene and Tropical Medicine, London, UK; 4 Central Clinical School, Monash University, Clayton, Australia; 5 School of Public Health, Georgia State University, Atlanta, GA USA; 6 Institute for Global Health and Infectious Diseases, University of North Carolina at Chapel Hill, Chapel Hill, NC USA

**Keywords:** HIV, Youth, HIV self-testing, Nigeria, LMICs

## Abstract

**Background:**

HIV self-testing (HIVST) provides young people with a convenient, discreet, and empowering way to know their HIV status. However, there is limited knowledge of young people’s preferences for HIVST services and potential factors that may influence the uptake of HIVST among this population. The purpose of this research was to use qualitative methods to examine HIVST preferences among Nigerian youth.

**Methods:**

Semi-structured in-depth interviews with a purposive sample of young people 14–24 years old were conducted in Lagos, Nigeria. Data were analyzed thematically to identify themes and domains related to preferences and factors influencing the use of HIV self-testing.

**Results:**

A total of 65 youth with mean age of 21 years, were interviewed, and the majority were females (56%). Four themes emerged as the most important characteristics that may influence young people’s preferences for HIV self-testing: 1) Cost (i.e. majority of participants noted that they would pay between NGN500 to NGN1,500 naira (USD1.38–USD4.16) for oral HIV self-testing kits); 2) Testing method (i.e. although blood-based sample kits were more popular than oral-based self-testing kits, most preferred the oral-based option due to its perceived benefits and for some, phobia of needles); 3) Access location (i.e. participants suggested they preferred to obtain the HIVST kits from youth-friendly centers, pharmacies, private health facilities, and online stores); and 4) Continuing care and support (i.e. participants highlighted the importance of linkage to care with trained youth health workers for positive or negative test results or toll-free helpline).

**Conclusion:**

HIV self-testing preferences among Nigerian youth appear to be influenced by several factors including lower cost, less invasive testing method, location of testing, and linkage to care and support post testing. Findings underscore the need to address young people’s HIV self-testing preferences as a foundation for implementing programs and research to increase the uptake of HIVST.

**Supplementary Information:**

The online version contains supplementary material available at 10.1186/s12889-020-10072-1.

## Background

HIV testing is a key entry point to HIV prevention and treatment and efforts to achieve the first 95% of the Joint United Nations Programme on HIV/AIDS (UNAIDS) 95–95-95 targets, to ensure that 95% of people living with HIV know their status by 2030 [1, 2]. However, in Nigeria, only 3.8% of males and 4.0% of females ages 15–19 have ever tested for HIV [3]. Multi-level barriers at the individual (fear, low perception of risk), social (limited peer and social support), and structural (stigma, lack of testing sites) levels limit HIV testing among Nigerian youth [4–7]. Closing the testing gap will require innovative approaches to delivering HIV testing services that address these barriers, including targeted approaches that can reach young people who may not have otherwise tested.

In 2016, the World Health Organization (WHO) recommended HIV self-testing, as an alternative to traditional HIV testing services (HTS) given its potential to expand HIV testing access to the hardest to reach population [8]. Hard to reach population include young people who are at risk for or with an undiagnosed HIV infection who may not otherwise receive testing from conventional services [8]. HIV self-testing (HIVST) allows individuals to collect their sample (either oral- or blood-based), conduct the test, and interpret the results privately or with someone that they trust [9, 10]. More recently, HIVST was incorporated into the revised National HIV and AIDS strategic framework 2019–2021, as a priority policy and programmatic approach to HIV response in Nigeria [11]. Despite compelling evidence on the beneficial effects of HIVST [12], HIVST uptake remains limited among Nigerian youth, [13–16] raising concerns about missed opportunities to actively engage this population. Consequently, it is vital to understand young people’s preferences for HIVST, including factors that may facilitate or hinder uptake in Nigeria.

While evidence about the acceptability and feasibility of HIVST among young people [17–20] in Sub-Saharan Africa are emerging, the majority of these studies are quantitative in nature [12, 21–23], which limits our ability to truly understand their preferences for HIVST. Therefore, the purpose of this study is to contribute to the literature by listening to the voices of young people in a resource-limited setting (Nigeria) and highlight both their preferences and factors that may influence their uptake of HIVST. The ultimate goal is to identify and inform the development of evidence-based programs to increase uptake of HIVST among Nigerian youths.

## Methods

### Study design and procedures

We conducted semi-structured individual in-depth interviews with young people between the ages of 14 to 24 years in October 2018. The study took place at the Nigerian Institute of Medical Research, Lagos Nigeria. The participants were purposively recruited to ensure a balance of male and female respondents and a broad distribution of participants across the age spectrum. Young people were eligible to participate in this study if they were between the ages of 14–24 years and were able to provide informed consent. The sample size for this study was set to reach saturation with regards to the emerging themes from the interviews [24]. The research team recruited participants from technical colleges, institutional campuses, and open community settings. As potential participants were approached, the research team member explained the purpose of the study, nature of the study and informed consent form. The length of the in-depth interview typically varied between 30 and 45 min. The interviews were conducted by trained interviewers, in a closed room at a convenient location. The interview sessions were audio-recorded and were conducted in English. During the interview, the USD2/OraQuick® HIV Self-Test kit (the only WHO pre-qualified product in Nigeria) and a pictorial demonstration on how to use the HIVST kit were shown to the participants but was not offered for testing. Participants were provided an incentive in the form of a phone recharge card voucher with a value of 1000 Naira (equivalent to USD3.26) to compensate for their time.

### Interview guide

The interview guide (Additional file 1) was pilot tested among ten young people aged 14–24 years and refined based on feedback from the pilot participants to fit the local Nigerian context. The semi-structured interview guide explored 1) individual’s sociodemographic characteristics; 2) experiences with HIV testing; 3) motivations and barriers to HIV testing; 4) perceptions of HIVST; 5) willingness to pay for HIVST; 6) HIVST delivery channels; 7) other services and support tools; and 8) linkage options following HIVST. Based on the literature, we employed a conceptual model to identify service characteristics at three different levels: individual, test and service delivery levels [25]. At the individual level, the questions were focused on experiences with HIV testing (i.e. HIV knowledge, testing history) and perceptions of HIVST. At the test level, the questions were focused on preferences regarding HIV testing modalities (i.e. oral- vs blood-based self-testing) and willingness to pay for HIVST. For the service delivery level, previous testing experiences, HIVST delivery channels, and other services and support tools including pre-and post-test counseling options, types of supporting information (i.e. hotline, videos, pamphlets or secure Apps) and willingness to do additional confirmatory testing, if positive, were explored.

### Data analysis

The responses from the in-depth interviews were transcribed verbatim in English. To avoid imposing participants’ responses on existing theories or frameworks, the research team used an inductive thematic analysis to guide the coding process and identified emerging themes as described by Virginia Braun & Victoria Clarke [26]. Two authors (CO and UN) individually conducted the initial close reading of all the transcripts to familiarize themselves with the data. Each of the transcripts was then manually coded using a pre-defined codebook which included a description and an example of each code. The coders met regularly to establish agreement related to the code definitions and quotations. Codes generated were compared across the two coders for differences and similarities and to evaluate inter-coder reliability. Discrepancies were resolved through team discussions. Codes were grouped into categories based on three domains (individual level, test level, and service delivery level characteristics) and factors likely to influence HIVST uptake. Themes were generated based on the interpretation of underlying meanings of the categories with relevant and illustrative quotes grouped and synthesized. We employed Guba’s qualitative trustworthiness criteria during and after the inquiry and throughout the coding process [27]. To ensure trustworthiness, an audit trail of the process of coding and thematic analysis was maintained and we organized periodic debriefing sessions with the larger research team. The Consolidated criteria for reporting qualitative research (COREQ) were followed to ensure quality in reporting the study [28].

### Ethical approval

Prior to participating in the study, participants were provided with detailed information on the study objectives, as well as potential risks and benefits. Written informed consent was obtained from all the study participants. Each participant was given a unique number, with which they were identified during the in-depth interview session, to preserve confidentiality. Ethical approvals for the study were granted by the Saint Louis University and the Nigerian Institute of Medical Research Institutional Review Boards.

## Results

### Participants characteristics

Of the 65 in-depth interview participants, 56.9% were females (Table 1). The participants were between the ages of 14 to 24 years, with a mean age of 21.2 years (SD = 2.6). More than half of the participants self-identified as Yoruba (60.0%) whereas 26.2% self-identified as Igbo and 12.3% self-identified as belonging to other ethnic groups in Nigeria. Nearly all the participants were single (93.0%), Christian (72.3%) and had obtained a secondary education (81.5%). About 72.3% of the participants reported having tested for HIV in the past.

**Table 1:** Demographic characteristics of the participants in the qualitative study, Lagos, Nigeria, 2018

	n	%
**Total**	65	100.0
**Age, years mean (SD)**	21.2	(2.6)
**Sex**		
Male	28	43.1
Female	37	56.9
**Marital Status**		
Single	61	93.8
Married/Engaged	2	3.1
**Ethnic Group**		
Yoruba	39	60.0
Igbo	17	26.2
Others	8	12.3
**Religion**		
Christian	47	72.3
Muslim	15	23.1
Others	1	1.5
**Highest Level of Education**		
Primary	5	7.7
Secondary	53	81.5
Higher than secondary	3	4.6
**Occupation**		
Employed	2	3.1
Self-employed	2	3.1
Unemployed student	57	87.7
**Ever Tested for HIV**		
Yes	43	66.2
No	17	26.2
**Ever Heard of HIV Self-testing**		
Yes	2	3.1
No	63	96.9

Note: Some frequencies do not add up to the total due to missing observations

### Individual level characteristics - perceived facilitators of HIV self-testing

In terms of awareness of HIV self-testing (HIVST), the majority of participants were unaware of HIVST (96.9%), specifically the oral-based self-testing kit (which was undergoing regulatory approvals in the country at the time of the interview). Although the concept of self-testing for HIV was relatively new to most of the participants, the majority were open to using it. Participants had positive responses towards HIVST after a sample test kit was shown to them, which included a detailed explanation of the testing process by the interviewer. Generally, most participants valued their ability to take control of their health with little or no external opinions or reputes, except for a few who were outwardly opposed to using HIVST. Most participants emphasized the perceived easiness of using the HIVST kit and a few noted its similarities with other home test kits including pregnancy test kit, as described below:

If we had an HIV testing kit like a pregnancy test, it would be very good because I could just get a kit, do it in the confines of my room and then nobody’s judging me, nobody’s giving me this look, like “Maybe she’s done something. Why does she want to know her status?” (#65, Female, ever tested for HIV)

The HIVST kit was often highlighted by the participants as a measure for reducing stigma and discrimination around testing for HIV, as described below:

“You could walk into any pharmacy or store and get it [HIVST kit], that's like the best thing that could happen right now because it reduces the chances of discrimination and stigmatization. Because in the society that we live in right now, even without people knowing your actual status, they get to discriminate just because you're getting tested in the first place, and that alone brings fear to the minds of people.” (#65, Female, ever tested for HIV)

Accessibility and timing were viewed by the respondents as key decision points for using the HIVST kit against facility-based testing, noting the ability to conduct the HIV test within the confines of the home, which saves the time of going out to the clinic and avoiding long wait times:

“this is something [HIVST] that I could buy at the pharmacy when I want and I can do it within a confined space… it will also save me the stress of going to the hospital or waiting for one of those tents to be set up at an awareness event whenever they decide to. It gives me convenience.”

(#08, Male, never tested for HIV)

One participant noted how this testing modality may reach most young people and enable them to know their status when introduced in Nigeria:

“if you create the kits, it will make more people come out to check for their status, and then prevent [HIV] too because if people do not know their status, they won't be able to get the necessary treatment. So, if I could check it myself, I definitely could get the right treatment for it”. (#61, Female, ever tested for HIV)

Some understood HIVST as an alternative form of prevention (i.e. condom use) against sexually transmitted infections (STI), as two participants explained the perceived benefit of using the HIVST kit before every sexual encounter:

“let's say for a girl and boy, they're about to have sex if you meet a girl in a night club and ask what is your HIV status, you already seem unsure but this is very fast and you can use this to know if the person is HIV positive” (#02, Male, ever tested for HIV)

“before you have sex with your partner, at least you may say, “Okay, let’s just do this [HIVST]. Let me feel comfortable.” And show, the HIV test result first” (#21, Female, ever tested for HIV

### Individual level characteristics - perceived barriers to HIV self-testing

Few participants who expressed mixed feeling towards HIVST highlighted that the absence of post-test counseling or follow-up care was a major concern because they believed that it could lead to suicide or personal harm after knowing the results, as opposed to testing at a health-care facility where counselors and doctors are present onsite for follow-up consultations, immediate treatment, and support:

“[after using the HIVST kit] if he/she finds out they have HIV, number one the person may not tell anybody and then the person may go into depression and maybe start doing drugs or doing other things or this person may just go and kill himself.” (#10, Female, never tested for HIV)

“Are there any directions? I don't mean how to use it. If I test and I don't commit suicide, how will I get medical advice? Do I just walk to the hospital and tell them? Because I mean that is very hard to just walk into the hospital and tell them.” (#02, Male, ever tested for HIV)

A select number of participants questioned the accuracy of the HIVST result, noting that the process “*seems very quick and almost too good to be true” (#01, Male, ever tested for HIV).* Alternatively, some would prefer going to the clinic to test because they believed that the blood-based HIV test done at the clinic or by a health professional gives a more accurate result. A participant explained:

“I prefer going to the hospital … Because I would know much better. I don’t know if the kit would show me the correct result, or not.” (#64, Female, ever tested for HIV)

A minority of the participants stated their concerns about the inability to properly use the HIVST kit and interpret the results without any errors or adverse events. A participant noted that there might be difficulties in using the kit if there is low literacy level, noting that:

“I just feel as though a professional should be there … It just seems as if there should be at least a level of know-how in doing it [HIVST], I just feel like somebody might get themselves injured or maybe go about it the wrong way” (#08, Male, never tested for HIV)

### Test level characteristics – costs, testing method and nature of packaging

The cost of the HIVST kit was identified as a strong determinant for choosing HIV testing services. Although most prefer the cost of the kit to be around 500 Naira (approximately USD1.38) to 1,500 Naira (approximately USD4.15), a few suggested costs as low as 200 Naira (approximately USD0.55) and as high as 4,000 Naira (approximately USD11.07). Some argued that if the HIVST kit were to be sold at an unaffordable cost, most young people might not be willing to purchase the kit because some hospitals and non-governmental organizations (NGOs) provide testing for free or at a subsidized rate:

“If it's overly expensive most people would not want to patronize it because getting tested right now at some facilities is free. In some facilities, it is as low as 500 naira or 1,000 naira in Nigeria right now. So, if it's not so expensive, people would prefer this [HIVST] than walking into facilities” (#65, Female, ever tested for HIV)

The oral-based HIVST was preferred by most of the participants when compared to the blood-based HIV test conducted by a health worker or tester, due to fear of pain and discomfort from the needle pricking. Some participants shared the following:

“Most people, like myself, have a phobia of needles and even going to hospital to get treatment and all that, they don't really like the idea of injections. For example, my cousin, would rather take any medication than to go to the hospital to get pricked by a needle. So, I think I'll prefer using a swab in the mouth to test for HIV..” (#65, Female, ever tested for HIV)

“you won't have to stress yourself going to the hospital, injecting a needle in you to drain out your blood. And after that, you will still be waiting for the results to come out.” (#37, Female, ever tested for HIV)

Other related discussions around test characteristics included the presentation and packaging of the HIVST kit. Some felt that the branding of the kit was medical-looking, subdued and could be rebranded to look more youth-friendly and discreet:

“So it’s too naked and it doesn’t say much … it’s not something that I want to buy to use. It’s too, can I use the word vague? I think there needs to be words on the packaging. Add some colors!” (#02, Male, ever tested for HIV)

Whereas, few preferred *a total package* in the form of a prevention box that will include the HIVST kit, condoms, pregnancy test kit, lubricants, other STI test kits, malaria, and tuberculosis test kits:

“if there were kits that included something to test for an STI and something to test for HIV as well, that works … they could make it a total package and even have a pregnancy in there as well…” (#65, Female, ever tested for HIV)

“I would also like tuberculosis [kit]. It has to be inside [the box] because tuberculosis and HIV kind of go hand in hand sometimes”. (#26, Female, ever tested for HIV)

### Service delivery level characteristics – testing experiences, access locations, continuing care and support

Among those who previously tested for HIV, the majority described their past testing experiences and the attitudes of healthcare workers as another important deterrent from testing at the hospital and thus the preference for wanting to use the oral HIVST kit. For some, the lack of compassion between the tester and the patient and fear of test result manipulation at health facilities, due to lack of provider-patient relationship and trust, were mentioned as influential factors that drive preferences for HIV testing options. For those who had never tested, the most frequently cited reason for choosing the oral-based HIVST was the issue of cross-infection related to the multiple-use of disposable needles while using the blood-based HIV testing at traditional testing venues:

“Because maybe I will get infected since they are going to use a needle and inject me, so there is fear that the needle will get infected … I am scared that [the needle] it’s not sterile … ” (#46, Male, never tested for HIV)

In terms of location to access the HIVST kits, privately-owned, registered pharmacies, youth-friendly centers, supermarkets, and online stores were the most cited locations. Participants generally associated public, government-owned facilities with less accurate HIV test results and low-quality settings; whereas private health facilities were associated with more accurate HIV test results and high-quality settings.

Specifically, few participants highlighted that they would rather obtain the HIVST kit from privately-owned, registered pharmacies than patent medicine vendors (also known as a chemist in Nigeria), to guarantee the quality of the test kit and control against counterfeit HIVST kits:

“Pharmacies could sell it as well, but not these, kind of small, small, pharmacies, like chemist shop, I mean well-recognized pharmacy should sell this Oraquick HIV test.” (#51, Female, ever tested for HIV)

Respondents valued a range of mediums for receiving supporting information to complete the HIV test, including a step-by-step guide on how to conduct the test and pre-and post-test counseling information. There was a slight preference for online video tutorials on how to use the HIVST kit and culturally-adapted pamphlets with graphic images, cartoons and minimal texts translated into three main Nigerian languages. For linkages to appropriate care and support after testing, most favored receiving post-test counseling from a younger health worker and a readily available toll-free helpline number for follow-up questions and linkage to the nearest health facility:

“if there's like a number or a helpline, maybe toll-free. I prefer toll free so I can talk to the person and the person still gives me some insight on how to go about it…” (#02, Male, ever tested for HIV)

“Maybe a general healthcare line that a person could call and then be referred to a close healthcare center around your place” (#65, Female, ever tested for HIV)

Motivations to seek confirmatory HIV test in the event of a positive result on the HIVST included, encouragement from peers, family members or healthcare worker, denial about the initial test result, lack of satisfaction of test result and the possibility of living longer under treatment and care:

“As a young person I would want to live as long as I can with the disease so that alone can spur me to want to go for confirmation and also the slightest chance that maybe the first test was wrong in the hope, probably would spur me to go for the confirmation test” (#01, Male, ever tested for HIV)

“Because I might not be that satisfied that I'm positive, first time using the HIVST, but I might think maybe it's not true, maybe it's giving me wrong details.” (#45, Male, ever tested for HIV)

Lastly, participants valued self-testing done privately or with a trusted individual compared to group (pooled) self-testing, to avoid a breach of confidentiality or invasion of privacy, as described below:

“I prefer one-on-one [HIV testing] rather than test in cliques … ” (#54, Female, ever tested for HIV)

“Most people would just prefer to do it [HIV self-test] privately. I think I would prefer to do it privately, and then if it comes out positive or negative, I'll determine who I get to tell or who should know about it” (#65, Female, ever tested for HIV)

## Discussion

The present study examined young people’s preferences, as well as factors likely to influence the uptake of HIV self-testing among young people in Nigeria. Numerous studies have explored the acceptability and feasibility of using HIVST among young people in sub-Saharan Africa, but only a few studies have focused on young people’s preferences for HIVST service delivery [20, 21, 29]. In-depth exploration of preferences including individual-, test- and service delivery-level characteristics, is an important approach to optimize the design, implementation and effectiveness of HIVST services for young people. Findings from this study can inform strategies to increase the uptake of HIVST as an alternative to facility-based testing services, particularly among at-risk populations who may not have tested otherwise. Although a substantial majority of participants (~ 97%) were unaware of HIVST before the study, participants had a relatively positive overall view of HIVST compared to facility-based testing. Consistent with studies conducted among similar population groups in sub-Saharan Africa [19, 20, 30], the potential benefits of HIVST included ease of use, accessibility, and empowerment of young people.

Test level characteristics were described as important factors that may influence young people’s preferences for HIVST. Comparable to other studies [21, 31, 32], costs, testing methods, and nature of packaging emerged as test-level factors that influenced young people’s preferences for HIVST. Although, few participants believed that a blood-based sample would accurately detect the virus, most preferred the oral-based sample due to fear of pain, discomfort and cross-infection from needle pricking associated with blood-based samples. Given young people’s limited access to financial resources, cost was a concern for the participants in choosing what services to use. These findings are consistent with an earlier study that was conducted among young people in Zimbabwe that suggest that young people’s low access to financial resources and strong aversion to price may deter uptake of HIVST [20]. Uptake of the HIVST kit may be impacted if the cost of purchasing the kit is high and should be given thorough consideration in the development of interventions and programs geared towards improving uptake of HIVST among young people. Although the advantages of using the HIVST kit were greater, most participants noted that they will rather pay less to test for HIV at a health facility. Several participants also reported that their negative past experiences while testing with a healthcare worker at the facility influenced their preferences for HIV self-testing, which was observed in other studies conducted in SSA [33–36].

To promote the uptake and performance of HIVST, special attention should be paid to the packaging and instructions for HIVST to ensure that they are tailored to young people’s needs and local literacy levels [37]. For instance, in our study, packaging of the test kit was suggested as an important factor that may influence participants’ decision to purchase the kit at a local pharmacy or supermarket. Some participants suggested repackaging the kits to be more discreet and youth-friendly, which includes adding colors and graphic designs. Study participants suggested that such designs would encourage uptake of HIVST kits among young people because it would be appealing to young people and could potentially reduce the stigma around HIV testing. In addition, participants were opposed to having lengthy and intricate texts in the instruction manual, as some testers may have low literacy levels or patience to read the instructions. As a result, there might be a possibility of experiencing errors in using the kit if the instructions are not properly illustrated or understood.

When considering service delivery characteristics of HIVST, the nature of access options and kit distribution method were suggested as important factors that may influence participants’ decisions to self-test. Private facilities were preferred over public facilities for the distribution of the HIVST kits. Public facilities were perceived to result in lower test accuracy, whereas, testing done at private facilities was believed to be more accurate. A few participants noted that by purchasing the kit from registered pharmacies, there would be a higher guarantee of the quality of the kit. The reasoning being that patent medicine vendors (defined as “a person without formal training in pharmacy who sells orthodox pharmaceutical products on a retail basis for profit” [38]) are often associated with the production of counterfeit drugs and medical devices in Nigeria [39].

Although the young people in our study expressed a preference for the oral-based HIVST in comparison to facility-based testing, concerns were raised regarding to the lack of pre- and post-test counselling and linkage to care. Such concerns have contributed towards the delay in expansion and adoption of HIVST in national HIV policies or programmes in low-income settings [40, 41]. Therefore, there is need to better understand pragmatic and innovative strategies to monitor HIVST use and facilitate linkage to care and support services. Preferred method for follow-up included leveraging digital tools, such as hotlines, and individual-level follow-up by a trusted individual or healthcare worker. Given the emergence of social media and technology, mHealth platforms has been shown to be acceptable among young people in other settings [42, 43] and may provide an avenue to monitor HIVST usage and linkage [40].

Taken together, our findings indicate that HIVST may have the potential to expand the reach of HIV testing to young people experiencing individual, social and structural barriers to testing. HIV testing services, including HIVST delivery, can be better tailored to fit the needs of these young people in Nigeria. In addition, findings from this study informed the development of a discrete choice experiment (DCEs) to further quantify the trade-offs between preferences for HIVST that young people in Nigeria are willing to make.

There are several limitations to our study worth mentioning. The population of this study may not be representative of young people in the entire country given that this study was conducted in a predominantly urban region of Nigeria. Therefore, results of this study should be interpreted with caution. Of note, this qualitative analysis was designed to generate nuanced understanding and identify unique characteristics around preferences for HIVST. The second limitation is that the majority of the participants had not seen or used HIVST before the study. Their responses were based on their interaction with the kit during the study and this may not be enough exposure to grasp the intricacies of using the kit. To mitigate this issue, the researchers responded to additional questions participants had about HIVST. While our study shows high acceptability of HIVST among young people, further studies are needed to explore the usability of HIVST among young people. Despite these limitations, this study provides timely and useful guidance for in-country implementers and policy makers who seek to expand the reach and uptake of HIV testing among young people in sub-Saharan Africa. While HIV testing has been extensively studied within this population group, this is among the first study, conducted in Nigeria, that describes preferences for HIVST services among youth who had tested previously for HIV and youth who had never tested for HIV.

## Conclusion

Our study findings indicate a high acceptance for the use of HIVST when made available in Nigeria, for reasons of self-empowerment, ease of use and accessibility. We delineate how the individual-, test-, service delivery level characteristics could influence the design, implementation and uptake of HIVST services in Nigeria. With effective strategies in place for linkage to post-test services following HIVST, there is a strong possibility that HIVST will be an appropriate approach to reach youth who may not otherwise test and maybe pivotal to achieve the first of the UNAIDS 95–95-95 targets —knowing one’s HIV status.

## Supplementary Information

**Additional file 1.** In-depth interview guide.

## Data Availability

The datasets generated and analyzed during the current study, including transcripts of the interviews, are not publicly available due to concerns regarding confidentiality and data protection but are available from the corresponding author on reasonable request.

## Abbreviations

HTS: HIV testing services
HIVST: HIV Self-testing
LMICs: Low and Middle Income countries
UNAIDS: Joint United Nations Programme on HIV/AIDS
SSA: Sub-Saharan Africa
WHO: World Health Organization
NIMR: Nigerian Institute of Medical Research
CO: Chisom Obiezu-Umeh
TG: Titilola Gbajabiamila
OE: Oliver Ezechi
UN: Ucheoma Nwaozuru
JJO: Jason J. Ong
II: Ifeoma Idigbe
DO: David Oladele
AZM: Adesola Z Musa
FU: Florida Uzoaru
CA: Collins Airhihenbuwa
JDT: Joseph D Tucker
JI: Juliet Iwelunmor
