# Supplementary material for: Young people’s preferences for HIV self-testing services in Nigeria: a qualitative analysis
Source: BMC Public Health. 2021 Jan 7;21:67. doi: 10.1186/s12889-020-10072-1 (PMC7792110; doi:10.1186/s12889-020-10072-1)
Supplement: Supplementary file 1 — Additional file 1. In-depth interview guide. [file 12889_2020_10072_MOESM1_ESM.docx]

**Innovative Approaches to Expand Youth-Friendly HIV Self Testing In-depth Interview Questions**

**Introduction**

Hello, thank you for volunteering to participate in this research study. This interview is a component of the Innovative Approaches to Expand Youth-Friendly HIV Self Testing research project. This research study is conducted through partnership with researchers at Saint Louis University, University of North Carolina-Chapel Hill and Nigeria Institute of Medical Research. The overall purpose of this interview is to identify young people’s experience and opinion on HIV self-testing services in Nigeria. It is designed to find out what young people in Nigeria think about being able to test themselves for HIV (“self-testing”). We know that many people use HIV testing services, but we also know that many people have not yet gone for a test or had their last test a long time ago. So, we are interested in finding out what people think about testing themselves and their preferences.

Please, keep in mind that there are no right or wrong answers. We are really interested in your insights and opinions. Also, you do not have to answer any question that you do not wish to respond to. Please, can you confirm that you have voluntarily agreed to participate in this interview and have signed the consent form? As a reminder, any information that you share during this interview is confidential and would not be shared with anyone outside the research team. As mentioned in the consent form, this interview will last for approximately 60 minutes.

I would also like to remind you that your name or any identifying information about yourself or anyone that you mention during this interview will not be associated with your responses. This interview will be digitally audio-recorded. The recording is made so that project staff can later carefully review what was talked about in the interview. The digital audio recording will be used to make a written transcript of the interview. The digital audio recording and transcripts will be labelled with a participant ID number only.

Before we begin, please can you tell me how you prefer I refer to you during this interview?

Do you have any questions before we begin?

TURN ON TAPE:

The date is ____ /____ /_________.
This is interview # ________-________-________.

Let’s begin with talking about your background.

**Personal Characteristics**

1. Can you tell me a bit about yourself?

***Probes:***

- Where did you grow up?
- How old are you?
- What is your highest level of education?
- What would you say is your main occupation?
- What ethnic group are you from?
- What is your marital status?
- What would you describe your religion as?
- Where do you currently live?
- What are your parent’s occupations?
- What are their highest level of education? (Both Mother and Father)

**Experiences with HIV-testing**

1. Please tell me what comes to mind when I say the word HIV.
2. Please tell me what comes to mind when I say the word sexually transmitted diseases.
3. Please tell me what comes to mind when you think about **HIV testing.**
4. Can you tell me whether you have ever tested for HIV?
   1. For those who tested: what was your experience like? (open question) … then go into your Q6 below.
   2. For those who have NOT tested: have you thought about testing before? (i.e. your Q5 below)

***Probes:***

- **If NO**: if you wanted to get an HIV test, where would you rather go?

***Probes****:*

- 1. Test on your own
  2. General practice
  3. Sexual health clinic
  4. Hospital out patient’s clinic
  5. Peer led community testing
  6. Other preference?

1. FOR THOSE WHO HAVE TESTED AND THOSE WHO HAVE NOT:

- Have you ever thought that you ought to get tested for HIV, but did not?
  1. What got in the way of you getting tested at that time?

***Probes:***

- - - 1. Personal situation, relationship status, etc.
      2. Fear or anxiety about the test result
      3. Fear, anxiety or discomfort with testing (didn’t want to be seen, etc.)
      4. Feeling judged or negatively labelled
      5. What family, friends, neighbors, may think
      6. Time and resources
      7. Access (inconvenient, don’t know where to go, etc.)
      8. Didn’t like organization or health care provider
      9. Didn’t want to know at that, wasn’t ready (fear of the unknown)
      10. Would make you change: how you thought about yourself, your whole life, etc.
      11. Social norms/social support
  1. What would make it easier for you to get tested?

1. FOR THOSE WHO HAVE TESTED:

- Where did you do the last test? When was that?
  1. What was the experience like? What did you like about it? What did you dislike?
  2. What made you get tested this most recent time? Did you plan it?

***Probes:***

- - - 1. Personal, situation, relationship status
      2. Triggering event, such as exposure, newly diagnosed STI or feeling unwell
      3. Fear, anxiety related to exposure
      4. Peace of mind
      5. Regular testing
      6. Easy access (convenient opportunity to test)
      7. New partner
      8. Feelings of responsibility
      9. Media message
      10. Other?

***“HIV Self-Testing is a type of HIV testing you can do by yourself. It allows you to collect your own test specimen, perform the test, and interpret the results in private, either alone or with guidance from someone you trust.”***

**HIV self-testing (HIVST)**

1. Before today, have you heard about HIV self-testing?
2. What do you think about HIV self-testing kit?

***Probes:***

1. Convenient
2. Privacy
3. Taking charge of one’s own health
4. What in your opinion are the potential advantages of using a self-testing kit?

***Probes:***

1. Privacy/ anonymity/ confidentiality
2. Personal empowerment / taking charge of one’s own health
3. Saves cost/ No fare to the clinic
4. No pricking/ painless
5. Saves time / no waiting in queues
6. What are the disadvantages?

***Probes:***

1. Difficulties/mistakes in performing the test or interpreting the results
2. Absence of counsellor when testing/ increased distress after a positive result / increased possibility of self-harm or suicide/ increased possibility of harming others
3. Testing others without their consent
4. Reduced chance of disclosure / enrolment in care
5. Production of fake or poor quality test kits
6. FOR THOSE WHO HAVE USED HIVST IN THE PAST:

- How did you feel about using the HIV self-test kits?

***Probes:***

- - 1. How many times did you use the HIV-self test kit?
    2. Which ones did you use?
    3. How did you access the kits?
    4. Where do you usually conduct the testing?
    5. When?
    6. What were the good things about using the self-test kits?
    7. What were some of the no-so-good things?
    8. Any difficulties of using the kits?
    9. Any difficulties in interpreting the results?
    10. What were the usual reasons why you chose to use HIV-self tests compared to facility-based testing?

1. What do think about a prevention pack/box that will include HIV self-testing kit and other Sexually transmitted infection testing kit?
2. What type of tests would you like to have (or see) in the prevention pack/box?
3. In your opinion, what are the most important factors that would make you choose to test for HIV by yourself using the prevention pack/box? Why?
   1. After that, which is the most important and why?
   2. And then?
   3. Which of these is least important to you?

(Allow participants to freely speak… but you may use the following probes – it will be important to distinguish which factors were spontaneously discussed by participants compared to the ones that you probed as per theme below.)

***Probes***

- 1. Privacy/anonymity
  2. Ease of accessing the kits i.e. don’t have to wait for appointment
  3. How clear instructions are to use the kit
  4. Accuracy (or lack of accuracy) of test
  5. How quickly you get your results
  6. Type of test (i.e. oral vs. blood-based)
  7. Cost of kit
  8. Recommendation from someone - your peers or health professionals or community leader?
  9. Other?

1. If the Government was not providing it for free, what is the maximum you would you be prepared to pay for such prevention pack?

***Probes:***

1. Over what range would you consider it reasonable to pay for a test? (minimum and maximum)
2. It is also possible to do these HIV and STI tests, at the clinic or hospital. However, the prevention pack will let you test on your own. **Which one do you think is better, using the prevention box or going to the clinics? And Why?**
3. Do you have a preference for where you would like to buy the prevention box? Why?

***Probes:***

- - - 1. HIV services (sexual health centre, specialized GPs, hospitals)
      2. Pharmacy
      3. Online
      4. Supermarkets
      5. Vending machine
      6. Community-based organizations
      7. other

1. What do you think would be most helpful or supportive for you before using the prevention box? And after using the prevention box?

***Probes:***

1. Instruction leaflets? What type of information?
2. Watching a video online?
3. Have someone show you how to use it? Who? (health professional, peer, other)
4. Access to a free hotline? Who do you want to talk to? (health professional, peer, other)
5. Other?
6. If your HIV self-test is positive, this will need to be confirmed with another test in a health facility. What are the most important factors that would make you choose to do an additional test to confirm your status (confirmatory test)? Why?
   1. After that, which is the most important and why?
   2. And then?
   3. Which of these is least important to you?

We have reached the end of the interview. Do you have any questions that you would like to ask me?

Thank you very much for the time you have spent in answering my questions today. Please remember that this information is all confidential. I have learnt a lot from our discussion here today and hope that the time has also been useful to you.
